# Abstracts from Foreign Journals

**Published:** 1901-10

**Authors:** M. P. Ravenel, S. J. J. Harger

**Affiliations:** Philadelphia, Pa.; Philadelphia, Pa.


### ABSTRACTS FROM FOREIGN JOURNALS.

### FRENCH.

UNDER THE DIRECTION OF M. P. RAVENEL, M.D., AND S. J. J. HARGER, V.M.D., OF PHILADELPHIA, PA.

CANINE DISTEMPER—EXPERIMENTAL VACCINATION. Phisalix sometime ago demonstrated in the guinea-pig a spontaneous infection due to bacillus, cultures of which are also very virulent to the dog. Introduced into the veins of the latter animal, this microbe often produced a meningo-encephalomyelitis; but, according to the dose and the virulence of the culture employed, the evolution of the disease was varied. Sometimes it was very acute and fatal in eight to ten hours; at times the course was more slow, and the disease assumed a gastro-intestinal form.

The general course of this experimental disease in the dog was similar to the natural affection called canine distemper. Phisalix found that the biological and morphological characters of this microbe were identical with the bacillus of Lignieres found in distemper, and by renewed efforts succeeded in isolating the latter microbe in the dog. It was obtained most easily from cultures made from the blood and organs of the dog before the period of secondary infection with other microbes; nevertheless, he has been able to separate it from accessory bacterial forms in the guinea-pig inoculated in the peritoneal cavity with the cephalo-rachidian liquid of the dog, and from this peritoneal liquid reproduced on bouillon a bacillus similar to the organism specific to the guinea-pig, excepting that the former was less virulent to the guinea-pig. These two organisms produce in the dog phenomena almost identical. Cultures of each organism in bouillon progressively attenuate with age; reinoculations of ordinary bouillons at the end of variable times produce cultures of diverse attenuations; passing the organism through the dog or guinea-pig restores its natural virulence.

*Vaccination of the Dog.* Phisalix has obtained in the dog and the guinea-pig a perfect vaccination against the microbe of the latter, the method employed being the subcutaneous injection of the attenuated culture. Two to three c.c. were used for a dog that still had its milk teeth. At the point of injection a slight swelling forms, which disappears in forty-eight hours; no general symptoms. When the culture is more virulent local abscess and a slight rise in temperature, but no grave symptoms, follow.

The first inoculation is made with a very attenuated culture whose local action is insignificant; then follow three or four vaccinations with cultures of increasing virulence. The immunity can then be tested by the intravenous injection of a virulent culture, canine distemper bacillus, or by cohabitation with an infected animal (dog); in some cases the mucous secretion was smeared around the nostrils. The vaccinated dogs were immune. Also, the inoculated animals were injected intravenously and resisted infection, while the check animals died or were seriously infected.

*En résumé*, young dogs inoculated several times with attenuated cultures resist natural and experimental infection; also, the practical application of the preventive (and possibly curative) treatment will render valuable services to breeders and owners of dogs —that is, the author has produced immunity against distemper in the dog with attenuated cultures of a bacillus natural infecting the guinea-pig. From his experiments the disease of the guinea-pig and distemper in the dog, although existing in two distinct species, must be, if not identical, at least very much analogous.—*Annales de Med Vet.*

TREATMENT OF STRONGYLOSIS IN SHEEP. Peters, in 1896, noticed frequent deaths in a herd of 400 sheep. Symptoms: Emaciation and symptoms of anæmia. At the autopsy the strongylus contortis was found in large numbers in the rumen and the abomasum.

*Treatment.* The administration of 10 centigrammes of picronitrate of potassium dissolved in 50 grammes of water. The effects of this drench were remarkable. Improvement is seen in a few days, and the entire herd was treated successfully.

Two years afterward the author met two outbreaks of verminous pneumonia, the strongylus filaria having been found in the lungs and the stomach at the post-mortem. Such agents as fumigations of phenic acid, tar, intratracheal injections of turpentine, animal oil, etc., failed. The author injected into the trachea 5 grammes of the above solution and gave internally 40 to 50 grammes. The result was remarkable.

In verminous pneumonia in cattle 150 to 200 grammes gave similar results. For the same indications picrate of potash was formerly recommended by Zurn.—*Annales de Med Vet.*

